# Transition of colistin dependence into colistin resistance in *Acinetobacter baumannii*

**DOI:** 10.1038/s41598-017-14609-0

**Published:** 2017-10-27

**Authors:** Ji-Young Lee, Eun Seon Chung, Kwan Soo Ko

**Affiliations:** Department of Molecular Cell Biology and Samsung Medical Center, Sungkyunkwan University School of Medicine, Suwon, 16419 South Korea

## Abstract

We recently demonstrated a high rate of colistin dependence in *Acinetobacter baumannii* isolates exposed to colistin *in vitro*. In the present study, we obtained a colistin-resistant (H08-391R) and colistin-dependent mutant (H08-391D) from a colistin-susceptible parental strain (H08-391). We found that the colistin-dependent mutant converted into a stable colistin-resistant mutant (H08-391D-R) *in vitro* after four serial passages without colistin. H08-391D and H08-391D-R were both found to harbor defective lipid A, as indicated by matrix-assisted laser desorption ionization-time of flight (MALDI-TOF) mass spectrometry analysis. Additionally, both contained an IS*Aba1* insertion in *lpxC*, which encodes a lipid A biosynthetic enzyme. Further, membrane potential measurements using the fluorescent dye 3,3′-diethyloxacarbocyanine iodide (DiOC_2_[3]) showed that the membrane potential of H08-391D and H08-391D-R was significantly decreased as compared to that of the parental strain, H08-391. Moreover, these mutant strains exhibited increased susceptibilities to antibiotics other than colistin, which may be attributed to their outer membrane fragility. Such phenomena were identified in other *A. baumannii* strains (H06-855 and its derivatives). Taken together, our study reveals that the colistin-dependent phenotype is a transient phenotype that allows *A. baumannii* to survive under colistin pressure, and can transition to the extremely resistant phenotype, even in an antibiotic-free environment.

## Introduction

*Acinetobacter baumannii* belongs to the ESKAPE group of pathogens (*Enterococcus faecium, Staphylococcus aureus, Klebsiella pneumoniae, A. baumannii, Pseudomonas aeruginosa*, and *Enterobacter* spp.), regarded as the most important bacterial species in clinical settings^1^. Infections due to multidrug-resistant (MDR) *A. baumannii*, especially carbapenem-resistant strains, are a burden on healthcare facilities owing to limited antibiotic options for treatment^2^. The MDR *A. baumannii* has been recently ranked as a bacterium that poses the greatest health threat by World Health Organization^3^. Currently, colistin is prescribed as the last resort for the treatment of MDR *A. baumannii* infections^4^, but colistin resistance in clinical *A. baumannii* isolates has been increasingly reported^5–7^. Although the current colistin-resistance rates are still relatively low, the emergence of colistin-resistant *A. baumannii* is a serious public health concern as it limits the therapeutic options for patients.

Colistin resistance in *A. baumannii* is known to occur via several mechanisms. One of these is the addition of 4-amino-4-deoxy-L-arabinose (L-Ara4N) or phosphoethanolamine (pEtN) to the lipid A moiety of lipopolysaccharide (LPS), which results in a decrease in the net negative charge of the bacterial outer membrane^8–10^. Colistin resistance has been shown to be regulated by the PmrAB two-component regulatory system^11^. Moreover, the loss of LPS due to mutations in the genes related to LPS biosynthesis, such as *lpxC*, *lpxA*, or *lpxD*, is also known to be associated with colistin resistance^12,13^.

Recent studies have revealed that colistin-susceptible *A. baumannii* has a unique capacity to develop colistin dependence after exposure to colistin^14–16^. Colistin-resistant subpopulations were reportedly obtained by culturing colistin-susceptible (but heteroresistant) *A. baumannii* on cation-adjusted Mueller–Hinton (CA-MH) agar plates containing more than 8 mg/L colistin. As expected, many of these colistin-resistant bacteria could grow well on MH agar plates with 10 mg colistin discs. Interestingly, however, some colonies surviving at colistin concentrations above 8 mg/L grew only near the colistin discs. Further, the patients with colistin-dependent strains showed higher 3- and 7-day treatment failure rates as compared with those without colistin-dependent strains^16^.

In the present study, we obtained a colistin-resistant subpopulation and colistin-dependent mutant from a colistin-susceptible parental *A. baumannii* strain by population analysis and disc diffusion assay. We found that the colistin-dependent mutants developed colistin resistance by serial passages in the absence of colistin. We performed the genotypic and phenotypic characterization of the colistin-resistant and colistin-dependent *A. baumannii* strains to evaluate the differences in their lipid A structures. Our results show that colistin dependence in *A. baumannii* may be a transient phenotype en route to acquiring resistance to colistin, and would be one of strategies to tolerate colistin until resistance is developed.

## Results

### Conversion of colistin dependence into colistin resistance during successive passages in colistin-free medium

**Figure 1:**
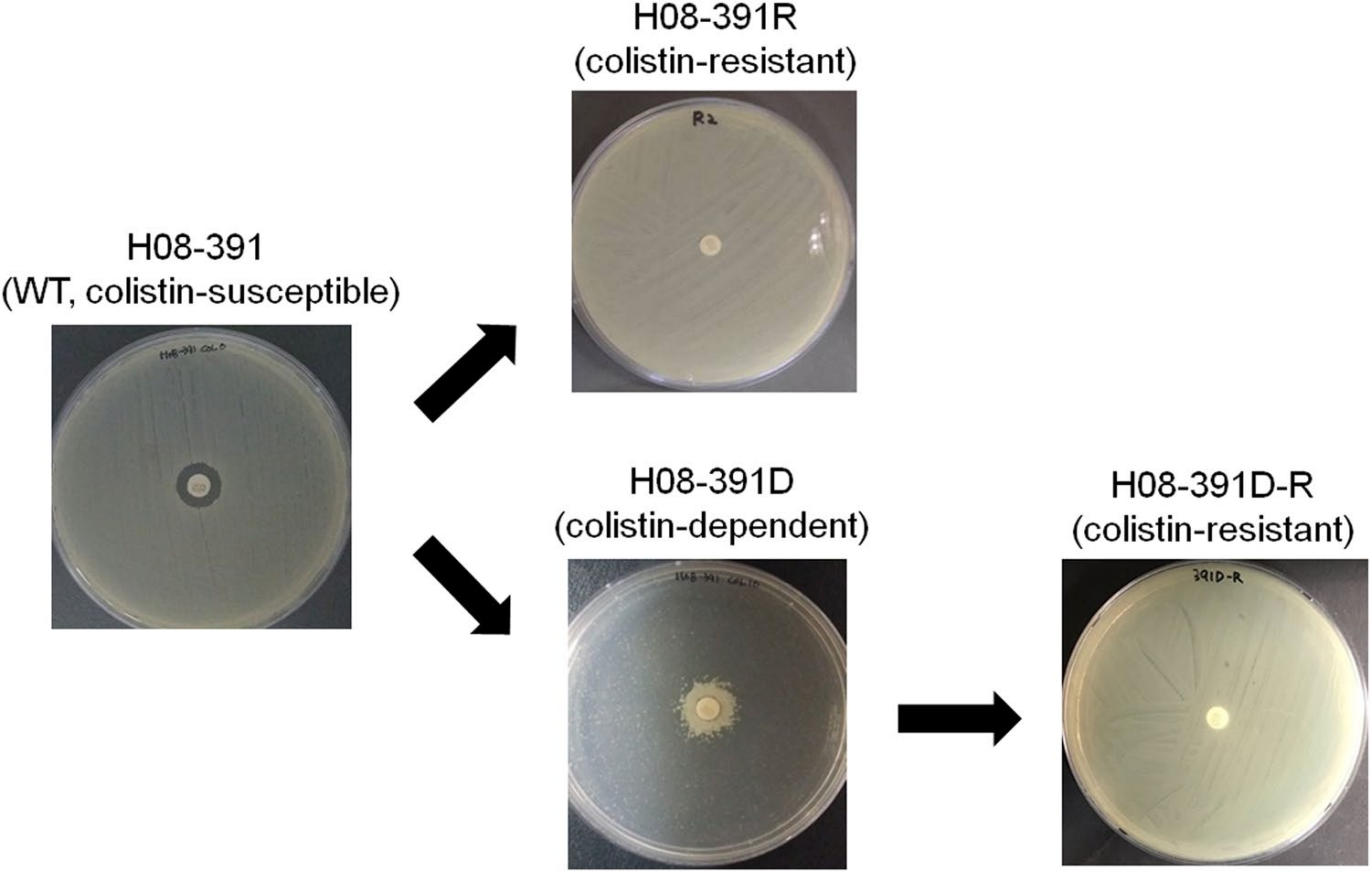
Colistin disc diffusion assays showing the development of colistin-dependent and -resistant mutants from the colistin-susceptible wild-type (WT) strain, H08-391. From a colistin-susceptible strain, H08-391, a colistin-resistant (H08-391R) and -dependent mutant (H08-391D) were selected *in vitro* using culture media containing 10 mg/L colistin. H08-391D-R was derived from the colistin-dependent mutant, H08-391D, through subsequent passages in the absence of colistin selection pressure, and exhibited the colistin-resistant phenotype.

We previously obtained colistin-dependent mutants from colistin-susceptible *A. baumannii* parental strains, including H08-391, through population analysis and colistin disc diffusion assay^16^. H08-391 strain belonged to ST75 based on Oxford scheme of multilocus sequence typing (MLST). To investigate the stability of colistin dependence in *A. baumannii*, we selected a colistin-dependent mutant, H08-391D, and a colistin-resistant subpopulation, H08-391R (Fig. 1). Their colistin minimum inhibitory concentrations (MICs) exceeded 64 mg/L. The colistin-dependent mutant H08-391D showed steady growth regardless of colistin concentration (up to 5120 mg/L). The phenomenon that a strain that survives at high antibiotic concentrations do not grow on a medium without antibiotics may be necessary condition of antibiotic dependence phenotype of the strain, but may not be sufficient. The resistant phenotype of the colistin-resistant mutant, H08-391R, was preserved during a number of successive passages in colistin-free medium. However, the colistin-dependent phenotype of H08-391D was completely converted into the resistant phenotype after four serial passages without colistin (Fig. 1). This colistin-resistant mutant, H08-391D-R, derived from the colistin-dependent mutant, exhibited a high level of resistance to colistin (MIC: 1280 mg/L), unlike that of the resistant mutant, H08-391R, derived from the susceptible wild-type (WT) strain (MIC: 64–128 mg/L). In addition, the colistin-resistant phenotype of H08-391D-R was stable for up to 20 serial passages in the absence of the antibiotic. Such conversion of the colistin-dependent phenotype into the colistin-resistant phenotype during successive passages in colistin-free media was also found in the strains: H06-855D and H09-146D (Fig. S1 in the supplemental material). Their parental strains H06-855 and H09-146 belonged to ST69 and ST75 in MLST analysis, respectively.

### Amino acid alterations in PmrCAB, LpxA, LpxD, and LpxC

To determine whether specific mutations in the genes associated with colistin resistance in *A. baumannii* conferred colistin dependence or resistance, the gene sequences for the *pmrCAB* operon (3575 bp), *lpxC* (903 bp), *lpxA* (789 bp), and *lpxD* (1071 bp) were determined in H08-391 and H06-855 and their derivatives (Table 1).

**Table 1:** Amino acid alterations in the PmrCAB, LpxA, LpxC and LpxD of the *A. baumannii* strains.

**Strain**	**Amino acid change in** ^**a,b**^ **:**											
	**PmrC**				**PmrA**	**PmrB**			**LpxA**		**LpxC**	**LpxD**
	CCP domain	Tryp_Spc domain			Res_reg domain	Unknown domain		HisKA domain	Unknown domain		Unknown domain	
	341	499	540	546	215	170	184	227	154	215	130	
*A. baumannii* ACICU		His	Asp	Ala	Arg	Pro	Glu	Ala	Gly	Arg		—
H08-391	25 aa insertion^c^	—	—	—	—		—	—		—	—	—
H08-391R	25 aa insertion	**Tyr**	—	—	**Gln**		**Lys**	**Val**		—	—	—
H08-391D	25 aa insertion	—	—	—	—		—	—		—	**IS** ***Aba1*** **insertion**	—
H08-391D-R	25 aa insertion	—	**Glu**	**Gly**	**Gln**		—	—		**Lys**	**IS** ***Aba1*** **insertion**	—
H06-855	25 aa insertion	—	—	—	—	—	—	—	—	—	—	—
H06-855R	25 aa insertion	—	—	—	—	**Tyr**	—	—	—	—	—	—
H06-855D	25 aa insertion	—	—	—	—	—	—	—	**Gly insertion**	—	—	—
H06-855D-R	25 aa insertion	—	—	—	**Gln**	—	—	—	**Gly insertion**	—	—	—

^a^Amino acid substitutions and insertions in the colistin-resistant or -dependent strains are in boldface and underline. Amino acids shared with *A. baumannii* ACICU are indicated by hyphen (—).

^b^The predicted domains are indicated as follows: CCP, complement control protein domain; Tryp_Spc, trypsin-like serine protease domain; Res_reg, response regulator receiver domain; Unknown, unknown domain; and HisKA, histidine kinase (phosphoacceptor) domain.

^c^The sequence of the 25-amino acid insertion in PmrC is YQIPENLKKKWCKDGECYDDILIDS.

Compared with the sequences of the reference strain, *A. baumannii* ACICU, 19 nucleotide substitutions, including an insertion of a 75 bp fragment, were found in the *pmrC* in all our strains sequenced. Most of these nucleotide substitutions were synonymous, and amino acid alterations were found in three sites in the trypsin-like serine protease domain of the PmrC C-terminal region: His499Tyr in H08-391R, and Asp540Glu and Ala546Gly in H08-391D-R (Table 1). Thus, all three amino acid alterations were identified in the colistin-resistant mutants. No amino acid substitutions were found in PmrC of the strains in H06-855 lineage. An insertion of 25 amino acids at position 341 of PmrC was found in all strains, including the colistin-susceptible WT strains H08-391 and H06-855, and this insertion has been previously reported in other *A. baumannii* strains, such as *A. baumannii* ATCC 19606^8^. This insertion may not affect PmrC, as it does not introduce any premature stop codons to halt protein processing, or a frameshift mutation in the protein. In the PmrAB two-component regulatory system, one substitution (Arg215Gln) in the PmrA of H08-391R, H08-391D-R and H06-855D-R, and two substitutions (Glu184Lys and Ala227Val) and one substitution (Pro170Tyr) were identified in PmrB of H08-391R and H06-855R, respectively. One substitution (Arg215Lys) was found in the H08-391D-R LpxA and an insertion of glycine at position 154 of LpxA was found both in H06-855D and H06-855D-R. The *lpxC* gene of H08-391D and H08-391D-R was disrupted by an insertion sequence, IS*Aba1* (1189 bp), at position 386. IS*Aba1* may interrupt the lipid A biosynthetic function of LpxC, also known as UDP-3-O-acyl-N-acetylglucosamine deacetylase. No amino acid substitutions were found in LpxD. The nucleotide sequences obtained in this study have been submitted to the GenBank database under accession No. MF541731 to MF541778.

### Expression of the *pmrCAB*, *lpxAD*, and *lpxC* genes

**Figure 2:**
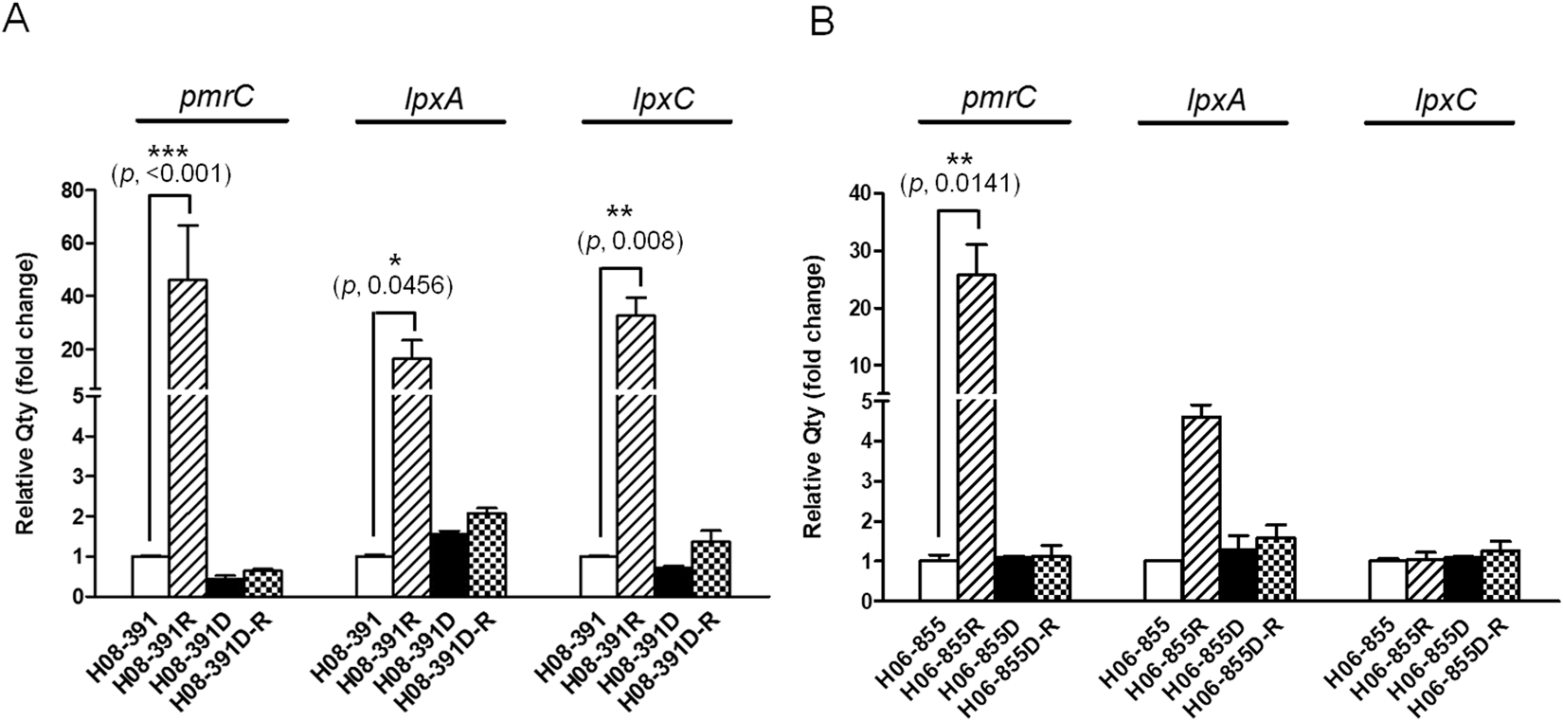
Expression levels of the *pmrC*, *lpxA*, and *lpxC* genes in the strains in *A. baumannii* H08-391 (**A**) and H06-855 (**B**) lineages. The three genes were found to be overexpressed only in the colistin-resistant mutants, H08-391R and/or H06-855R. Error bars represent the standard deviation of three biological repeats, each performed in duplicate. Statistical significance between each strain was determined using Student’s unpaired *t*-test. Data were analyzed using GraphPad Prism 5.

The *pmrCAB* operon is associated with the addition of pEtN to lipid A. We found that the expression level of *pmrC* was 46.1-fold higher in the colistin-resistant mutant, H08-391R, as compared to that in the colistin-susceptible parental strain, H08-391 (Fig. 2A). However, the expression levels of *pmrC* in H08-391D and H08-391D-R were not significantly different from those in the WT strain, H08-391. Likewise, the expression levels of *lpxA* and *lpxC*, which are involved in lipid A biosynthesis, were dramatically increased (16.3- and 32.5-fold, respectively) in H08-391R, as compared to those in H08-391 (Fig. 2A). Moreover, their expression levels in H08-391D and H08-391D-R were similar to those in H08-391. For H06-855 lineage, the expression level of *pmrC* was significantly increased in the colistin-resistant mutant, H06-855R by about 25.7-fold, compared to that in the colistin-susceptible parental strain (Fig. 2B). *lpxA* and *lpxC* showed no significant differences in their expression between colistin-susceptible WT strain H06-855 and its colistin-resistant or -dependent mutants, although H06-855R showed 4.6-fold higher level of *lpxA* expression than that of H06-855.

### Transcriptomic differences in colistin-susceptible strains and their colistin-dependent mutants

**Figure 3:**
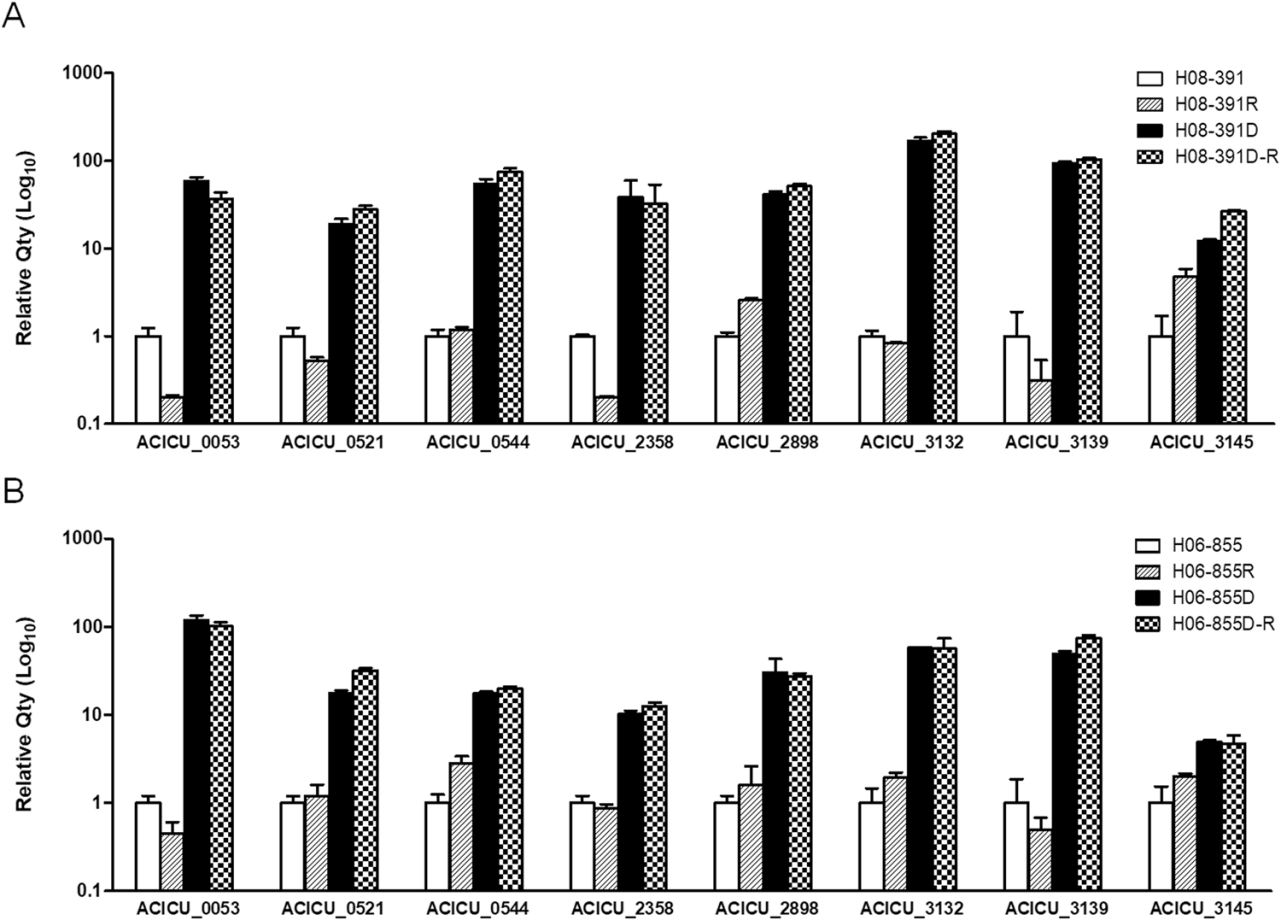
Genes showing high expression levels in colistin-dependent and its converted colistin-resistant strains. The expression levels were evaluated by mRNA sequencing. (**A**) H08-391 lineage; (**B**) H06-855 lineage. Error bars represent the standard deviation of three biological repeats, each performed in duplicate.

Transcriptomic analysis revealed that 14 genes exhibited more than 10-fold increases in expression in both colistin-dependent strains H08-391D and H06-855D compared with their colistin-susceptible WT strains (Table 2). ACICU_00544-00546 belonging to the same operon, which encodes a ATP-binding cassette (ABC)-type transporter system, showed higher expression levels in colistin-dependent mutants, exhibiting about 11- to 40-fold increases compared with that of colistin-susceptible strains. ACICU_02898 and ACICU_03145 encoding a lytic transglycosylase and a glycosyltransferase, respectively, also showed increased expression in colistin-dependent mutants, ranging from 14- to 37-fold. The expression of ACICU_00053, ACICU_03132 and ACICU_03329 encoding hypothetical proteins of unknown function were dramatically increased about 200- to 1700-fold in both colistin-dependent strains. Expression levels of eight genes among these 14 genes, showing more than 10-fold increased expression in mRNA sequencing, were compared between colistin-resistant and -dependent strains in H08-391 and H06-855 lineages by qRT-PCR. All tested genes showed increased expression levels consistently with mRNA sequencing results in colistin-dependent H08-391D and H06-855D strains and their derivated colistin-resistant strains (H08-391D-R and H06-855D-R) also have preserved increased expression of the genes (Fig. 3). However, colistin-resistant subpopulations, H08-391R and H06-855R, derived from colistin-susceptible strains, exhibited a low expression of these genes, unlike that of H08-391D-R and H06-855D-R, derived from the colistin-dependent strains (Fig. 3). These results suggest that highly expressed genes in colistin-dependent mutants may play a role in development of colistin-dependent mutants and up-regulation of these genes might be not directly involved in colistin resistance.

**Table 2:** Differentially expressed genes (more than 10-fold) in both colistin-dependent mutants (H08-391D and H06-855D) compared with their colistin-susceptible parental strains (H08-391 and H06-855).

**Locus_tag**	**Product**	**Operon**	**RPKM (Reads Per Kilobase per Million)**				**Fold change**	
			**H08-391**	**H08-391D**	**H06-855**	**H06-855D**	**H08-391D**	**H06-855D**
**Up-regulated gene**								
ACICU_00053	Hypothetical protein		38.667	16982.350	22.010	37062.303	**439.198**	**1683.918**
ACICU_00065	Hypothetical protein		82.529	1188.336	58.239	3712.802	**14.399**	**63.751**
ACICU_00305	Hypothetical protein		91.987	2368.679	62.165	2004.310	**25.750**	**32.242**
ACICU_00521	Hypothetical protein		35.500	355.880	39.004	240.859	**10.025**	**10.175**
ACICU_00544	Outer membrane protein	a	106.466	1193.712	68.466	1508.850	**11.212**	**22.038**
ACICU_00545	Peptide ABC transporter permease	a	98.025	1417.031	80.811	2586.167	**14.456**	**32.003**
ACICU_00546	Membrane-fusion protein	a	131.442	2034.333	110.587	4372.488	**15.477**	**39.539**
ACICU_02357	Hypothetical protein		201.592	10904.023	190.538	13381.578	**54.090**	**70.231**
ACICU_02358	Hypothetical protein		123.323	11859.916	111.913	17536.285	**96.169**	**156.695**
ACICU_02898	Soluble lytic transglycosylase fused to ABC-type amino acid-binding protein		61.690	834.879	72.603	1454.194	**13.533**	**20.029**
ACICU_03132	Hypothetical protein		40.038	8532.027	43.370	17674.980	**213.098**	**407.541**
ACICU_03139	Hypothetical protein		15.709	13786.089	17.672	24091.961	**877.584**	**1363.257**
ACICU_03145	Glycosyltransferase		9.883	190.734	6.414	236.188	**19.299**	**36.822**
ACICU_03329	Hypothetical protein		1422.636	32838.090	1101.933	37325.033	**23.083**	**33.872**
**Down-regulated gene**								
ACICU_00761	Trehalose-6-phosphate synthase	b	1070.680	39.526	3772.522	75.715	−**27.088**	−**49.825**
ACICU_00762	Trehalose-6-phosphatase	b	132.942	11.010	528.553	14.933	−**12.075**	−**35.395**
ACICU_01198	Ferredoxin		3971.377	100.454	12361.136	194.354	−**39.534**	−**63.601**
ACICU_01214	Hypothetical protein		812.757	30.218	1452.649	55.326	−**26.896**	−**26.256**
ACICU_01221	Hypothetical protein		904.150	53.892	6833.760	41.259	−**16.777**	−**165.631**
ACICU_01257	Hypothetical protein		5005.489	105.074	4975.259	176.541	−**47.638**	−**28.182**
ACICU_01420	Hypothetical protein		403.950	11.310	480.463	7.442	−**35.717**	−**64.557**
ACICU_01421	Hypothetical protein		285.098	13.422	494.538	16.113	−**21.241**	−**30.691**
ACICU_01422	Putative 17 kDa surface antigen		10458.164	89.390	103831.995	111.001	−**116.995**	−**935.416**
ACICU_01423	Hypothetical protein		61421.784	332.702	523433.243	451.036	−**184.615**	−**1160.513**
ACICU_01425	Hypothetical protein	c	1100.805	88.364	3043.597	183.081	−**12.458**	−**16.624**
ACICU_01426	Catalase	c	7499.909	348.235	19525.670	625.399	−**21.537**	−**31.221**
ACICU_01427	Dehydrogenase		1272.509	25.529	3654.288	42.142	−**49.845**	−**86.714**
ACICU_01429	Hypothetical protein		1522.708	58.514	23480.619	66.453	−**26.023**	−**353.342**
ACICU_02269	Hypothetical protein		304.781	21.541	642.656	37.335	−**14.149**	−**17.213**
ACICU_02276	Hypothetical protein		210.887	8.398	689.267	7.406	−**25.112**	−**93.063**
ACICU_02382	Hypothetical protein		335.294	18.735	502.657	41.647	−**17.896**	−**12.069**
ACICU_02406	Permease	d	1743.983	19.409	2894.196	97.369	−**89.853**	−**29.724**
ACICU_02407	Aspartate aminotransferase	d	3611.417	19.047	9451.849	131.564	−**189.605**	−**71.842**
ACICU_02431	Hypothetical protein		2448.491	56.370	8347.258	158.810	−**43.436**	−**52.561**
ACICU_02432	Glycosyltransferase	e	687.280	21.995	1721.156	40.976	−**31.248**	−**42.004**
ACICU_02433	SAM-dependent methyltransferase	e	787.885	25.007	2259.001	50.553	−**31.507**	−**44.686**
ACICU_02434	LmbE protein	e	849.278	19.039	2670.127	42.913	−**44.608**	−**62.222**
ACICU_02435	Putative acyl-CoA dehydrogenase-related protein	e	536.648	16.119	1664.439	33.717	−**33.293**	−**49.365**
ACICU_02436	Hypothetical protein		24595.952	178.724	92265.310	407.187	−**137.620**	−**226.592**
ACICU_02939	Hypothetical protein	f	955.159	30.215	3882.269	245.332	−**31.613**	−**15.825**
ACICU_02940	Hypothetical protein	f	1372.551	53.651	5114.186	358.084	−**25.583**	−**14.282**
ACICU_02941	Hypothetical protein	f	1078.157	43.149	3849.177	267.170	−**24.987**	−**14.407**
ACICU_02942	Hypothetical protein	f	421.248	15.412	1449.073	102.863	−**27.332**	−**14.087**
ACICU_02943	Hypothetical protein	f	748.425	35.505	2819.347	206.037	−**21.080**	−**13.684**
ACICU_02944	Putative hemagglutinin/hemolysin-like protein	g	1769.307	70.795	20053.928	1621.403	−**24.992**	−**12.368**
ACICU_02945	Hypothetical protein	g	2334.492	89.048	24804.562	1986.355	−**26.216**	−**12.487**
ACICU_03499	Hypothetical protein		1418.428	72.961	4635.150	122.217	−**19.441**	−**37.926**

In this transcriptomic analysis, 33 down-regulated genes (more than 10-fold) in both colistin-dependent mutants were also founded (Table 2). Most of the genes were annotated as hypothetical genes and only 12 genes encode proteins of known function.

### Lipid A modifications in the colistin-dependent and -resistant mutants

**Figure 4:**
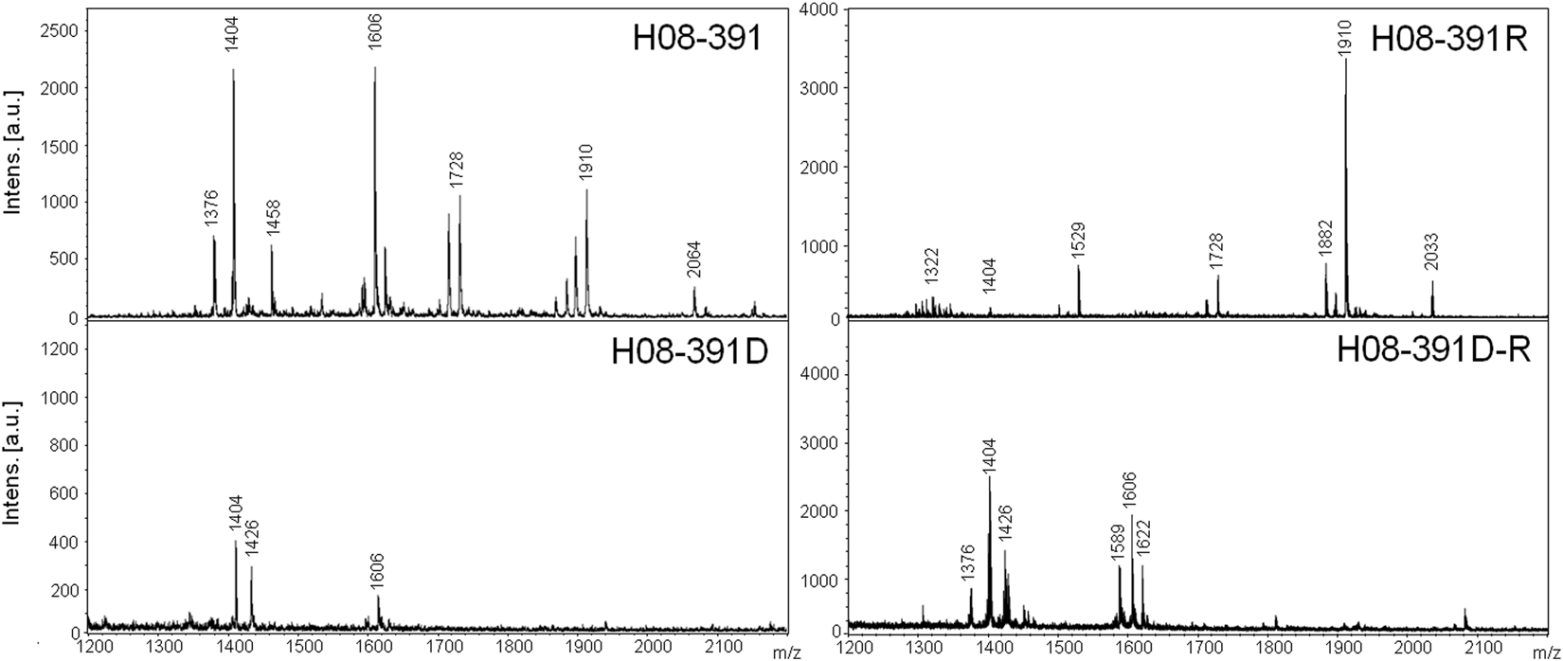
Negative-ion mode matrix-assisted laser desorption ionization-time of flight (MALDI-TOF) mass spectrometry spectra of the lipid A moieties of lipopolysaccharide isolated from the four strains: H08-391, H08-391R, H08-391D, and H08-391D-R.

We assessed the structural changes in LPS arising from differences in the lipid A structures between the colistin-resistant and colistin-dependent mutants. We characterized and compared the lipid A from H08-391 and its derivatives using matrix-assisted laser desorption ionization-time of flight (MALDI-TOF) mass spectrometry (MS). The mass spectrum for lipid A from the colistin-susceptible WT strain, H08-391, showed major peaks at the mass/charge ratios (*m*/*z*) of 1404, 1728, and 1910, corresponding to the tetra-, hexa-, and hepta-acylated lipid A species, respectively (Fig. 4). The lipid A from the colistin-resistant strain, H08-391R, showed major peaks at *m*/*z* 1728 and 1910, and an additional minor peak at *m*/*z* 2034, corresponding to the addition of one pEtN residue (Δ*m*/*z* = +124) to the hepta-acylated lipid A species. This pEtN-modified lipid A was only seen in the colistin-resistant strain, H08-391R, indicating that this modification was associated with colistin resistance in *A. baumannii*, and not with colistin dependence. Interestingly, all the major peaks, except for the peak at *m*/*z* 1404, completely disappeared in the mass spectrum for lipid A from the colistin-dependent strain, H08-391D. This suggests that the lipid A moiety in the colistin-dependent strain was either completely absent or had structural defects. The mass spectrum for lipid A from H08-391D-R suggests that the lipid A structure in this strain might be similar to that of its parental colistin-dependent strain, H08-391D. Although H08-391D-R was stable and exhibited higher colistin resistance compared to H08-391R, its lipid A structure differed completely from that of H08-391R, and was probably defective, like that of H08-391D.

### LPS deficiency in colistin-dependent *A. baumannii* H08-391D

**Figure 5:**
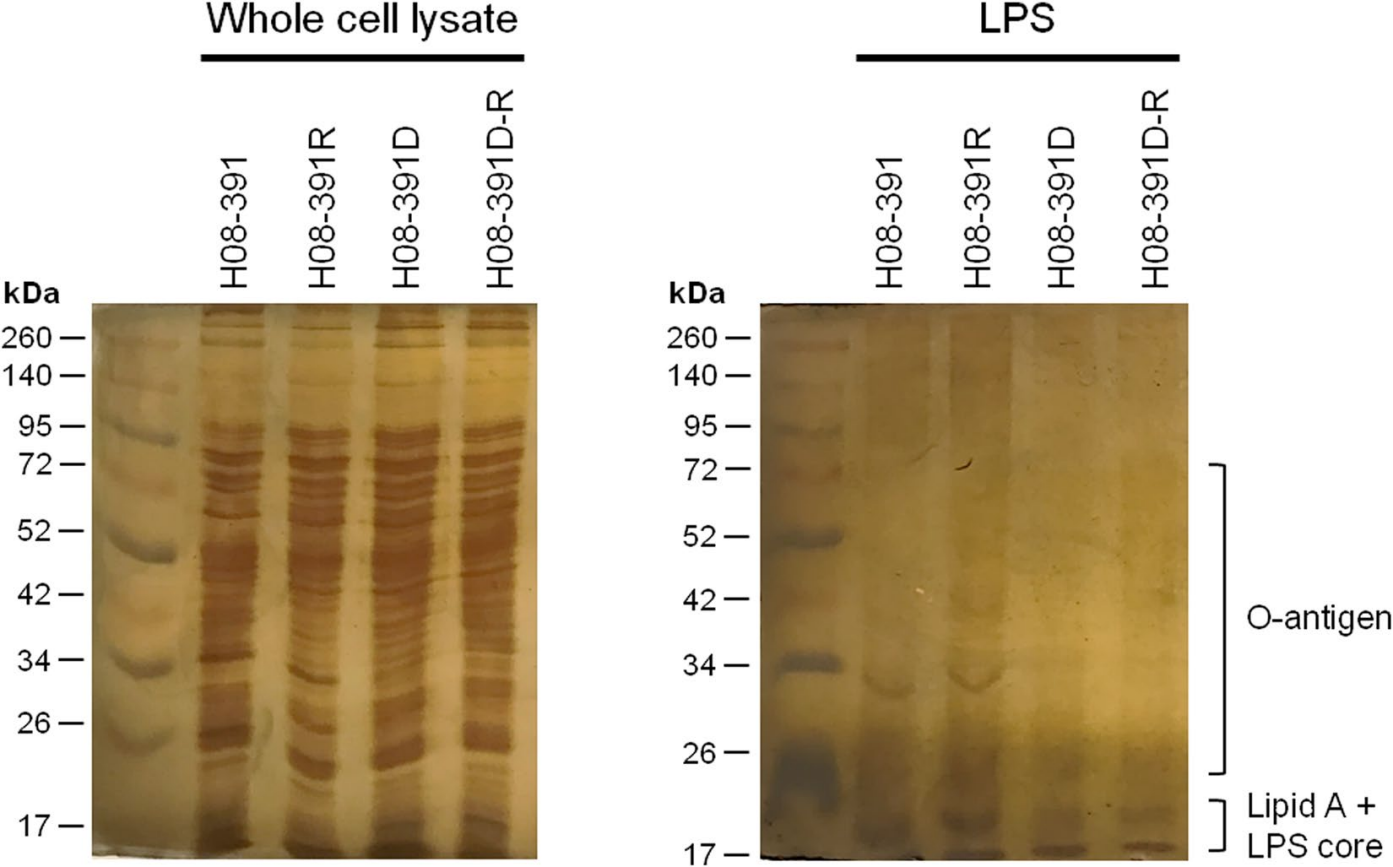
LPS profile of the strains H08-391, H08-391R, H08-391D, and H08-391D-R.

To assess the ability to produce LPS of colistin-dependent H08-391D with inactivated LpxC, we analyzed the purified LPS from each strain in the H08-391 lineage. To exclude the possibility a loss of LPS during the purification process, we also analyzed the whole cell lysates of each stain. All strains showed similar pattern in whole cell lysate but H08-391D strain, and its converted colistin-resistant strain H08-391D-R produced relatively small amounts of LPS compared with those of H08-391 and H08-391R (Fig. 5). In whole cell lysate, both H08-391 and H08-391R had the strong bands visible between 30 and 35 kDa corresponding to LPS core oligosaccharide with O-antigen. However, this band was not produced by H08-391D and H08-391D-R (Fig. 5). The band that may represent LPS core with O-antigen appeared in LPS purified in H08-391 and H08-391R, but not in H08-391D or H08-391D-R, consistent with the result shown in whole cell lysate. These data show that there are clear difference in the structure or quantity of the LPS between colistin-susceptible and -dependent strains.

### Differences in antimicrobial susceptibilities

To investigate the impact of lipid A modifications or deficiency on the antimicrobial susceptibilities of the mutant strains, we determined the MICs of selected antimicrobial agents for each strain. The colistin-susceptible WT strain, H08-391, was resistant to all antibiotics tested, except to polymyxins and tigecycline, while its colistin-resistant derivative, H08-391R, exhibited reduced susceptibility to polymyxins (MICs: 64–128 mg/L for colistin and 64 mg/L for polymyxin B, Table 3). The colistin-dependent mutant, H08-391D, showed increased susceptibility to several antimicrobial agents, including carbapenems. However, it exhibited an extremely high colistin MIC (>5120 mg/L). The converted colistin-resistant mutant, H08-391D-R, also showed increased susceptibility to most of the antibiotics tested. The colistin MIC (1280 mg/L) in H08-391D-R was also extremely high, despite being less than that of H08-391D (>5120 mg/L). As the dependent strain and its converted resistant strain were highly increased susceptibility to several antimicrobial agents, they displayed increase in sensitivity to the other antibiotics, such as vacomycin and azithromycin which are used to treat Gram-positive pathogens. These results were confirmed consistently in the strains of H06-855 lineage.

**Table 3:** Antimicrobial susceptibilities of the strains in *A. baumannii* H08-391 and H06-855 lineages to selected antibiotics.

**Antibiotic**	**Antimicrobial susceptibility (MIC, mg/L)** ^**a,b**^							
	**H08-391**				**H06-855**			
	**WT**	**R**	**D**	**D-R**	**WT**	**R**	**D**	**D-R**
Colistin	1 (S)	**64–128 (R)**	**>5120 (R)**	**1280 (R)**	1 (S)	**64 (R)**	**>5120 (R)**	**2560 (R)**
Polymyxin B	1 (S)	**64 (R)**	**>64 (R)**	**>64 (R)**	0.5 (S)	**64 (R)**	**>64 (R)**	**>64 (R)**
Imipenem	**>64 (R)**	**>64 (R)**	1 (S)	1 (S)	**16 (R)**	**16 (R)**	0.25 (S)	0.25 (S)
Meropenem	**>64 (R)**	**>64 (R)**	1 (S)	0.5 (S)	**16 (R)**	**8 (R)**	0.125 (S)	0.125 (S)
Tigecycline	2 (−)	2 (−)	0.125 (−)	0.125 (−)	4 (−)	2 (−)	0.25 (−)	0.25 (−)
Tetracycline	**>64 (R)**	**>64 (R)**	**16 (R)**	**16 (R)**	**16 (R)**	**16 (R)**	**16 (R)**	**16 (R)**
Ciprofloxacin	**>64 (R)**	**64 (R)**	**16 (R)**	**32 (R)**	**>64 (R)**	**64 (R)**	**32 (R)**	**32 (R)**
Amikacin	**128 (R)**	**128 (R)**	16 (S)	16 (S)	**>128 (R)**	**128 (R)**	2 (S)	2 (S)
Cefepime	**>64 (R)**	**>64 (R)**	2 (S)	4 (S)	**>64 (R)**	**>64 (R)**	4 (S)	4 (S)
Ceftriaxone	**>128 (R)**	**>128 (R)**	0.5 (S)	1 (S)	**>128 (R)**	**>128 (R)**	1 (S)	1 (S)
Ceftazidime	**>64 (R)**	**64 (R)**	1 (S)	2 (S)	**>64 (R)**	**64 (R)**	8 (S)	8 (S)
Piperacillin/tazobactam	**>256/4 (R)**	**>256/4 (R)**	**128/4 (R)**	**128/4 (R)**	**>256/4 (R)**	**>256/4 (R)**	**256/4 (R)**	**256/4 (R)**
Ampicillin/sulbactam	**64/32 (R)**	**32/16 (R)**	2/1 (S)	8/4 (S)	**>64/32 (R)**	**32/16 (R)**	4/2 (S)	4/2 (S)
Rifampin	2 (−)	1 (−)	≤0.06 (−)	≤0.06 (−)	4 (−)	2 (−)	≤0.06 (−)	≤0.06 (−)
Azithromycin	64 (−)	64 (−)	2 (−)	2 (−)	32 (−)	32 (−)	1 (−)	2 (−)
Vancomycin	>64 (−)	>64 (−)	1 (−)	1 (−)	>64 (−)	>64 (−)	2 (−)	4 (−)

^a^S, sensitive; R, resistant; −, no breakpoint.

^b^MIC values in bold and underline indicate resistance toward the antimicrobial agent.

### Impact of membrane permeability on colistin dependence and resistance

**Figure 6:**
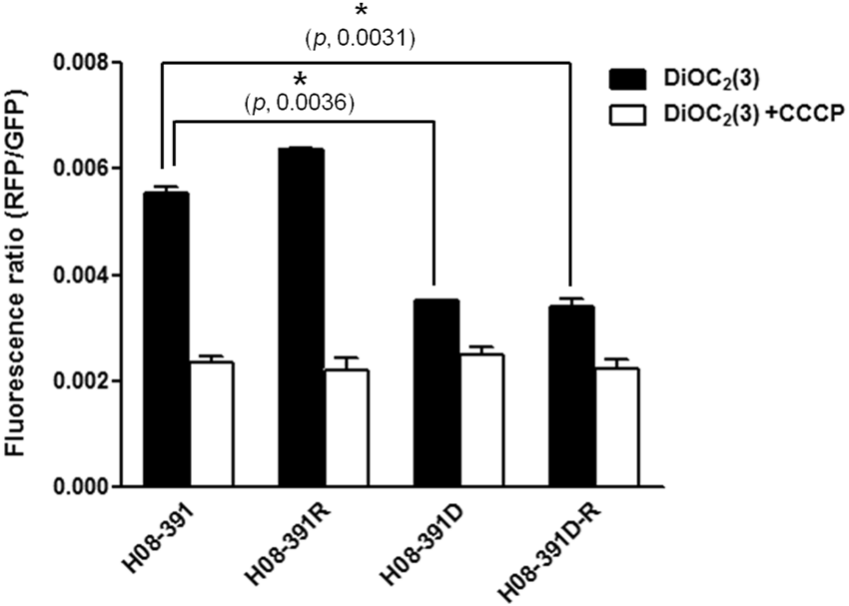
Comparison of the membrane potential in the strains H08-391, H08-391R, H08-391D, and H08-391D-R. Error bars represent the standard deviation of three biological repeats, each performed in duplicate. Statistically significant differences in membrane potential between the strains were analyzed using Student’s unpaired *t*-test using GraphPad Prism 5.

To establish a link between membrane permeability and the structure of lipid A, which resides in the bacterial outer membrane and affects colistin susceptibility, we measured the membrane potential of the strains using the fluorescent membrane-potential indicator probe, 3,3′-diethyloxacarbocyanine iodide (DiOC_2_[3]). There were no significant differences in the red to green fluorescence intensity ratios between H08-391 and H08-391R, indicating that the lipid A modification by pEtN in H08-391R had little effect on its outer membrane permeability (Fig. 6). However, the fluorescence intensity ratios in H08-391D and H08-391D-R were almost half of those observed in H08-391 and H08-391R. These results imply that the defective lipid A structures found in H08-391D and H08-391D-R significantly affected their membrane permeability. Upon cotreatment of the bacteria with DiOC_2_(3) and a proton ionophore, carbonyl cyanide 3-chlorophenylhydrazone (CCCP), the membrane potential decreased in all strains, indicating that the proton gradient was uniformly destroyed.

## Discussion

Several studies, including a recent study from our lab, have described the phenomenon of colistin dependence in *A. baumannii* ^14–16^. We reported that a substantial proportion (approximately one-third) of colistin-susceptible isolates developed colistin dependence after exposure to colistin^16^. Moreover, patients with *A. baumannii* bloodstream isolates that developed colistin dependence showed higher colistin-treatment failure than those without colistin-dependent isolates, although this result was not statistically significant. In this study, we demonstrated that colistin dependence develops into colistin resistance in the absence of antibiotic selection pressure.

The strain H08-391D showed the colistin-dependent phenotype, growing on agar plates only in the regions near the colistin disc. However, it exhibited a colistin MIC of >64 mg/L, as determined by the broth microdilution method, and this fell within the colistin-resistance range (>4 mg/L)^17^. H08-391D exhibited constant growth even at an extremely high colistin concentration of 5120 mg/L. However, this colistin-dependent phenotype was probably unstable, as it converted into the colistin-resistant phenotype after only the fourth serial passage in medium lacking colistin. The preservation of the IS*Aba1* insertion in *lpxC* or amino acid substitution of colistin-dependent mutants in their converted colistin-resistant mutants may indicate that the colistin resistance phenotype was derived from the colistin-dependent strains. Because the colistin-dependent strain lacks LPS, it could not be reversed into colistin susceptibility even on the media without colistin. Development of colistin resistance from colistin dependence may be a common phenomenon in *A. baumannii*, as it was exhibited by two other strains as well: H06-855D and H09-146D. These colistin-resistant mutants derived from the colistin-dependent mutants showed high-level resistance to colistin (MIC: 1280 mg/L). Further, their colistin-resistant phenotype was preserved for up to 20 serial transfers in the absence of selective pressure. This indicates that these mutants may be similar to the colistin-resistant mutant derived from the colistin-susceptible WT strain in terms of stability.

In gram-negative bacteria, including *A. baumannii*, colistin resistance has evolved owing to modifications in lipid A, which results in a decrease in the net charge of the outer membrane^8,9^. In addition, the loss of LPS owing to mutations or disruption of genes involved in lipid A biosynthesis, such as *lpxA*, *lpxD*, and *lpxC*, has been reported as responsible for colistin resistance in *A. baumannii* ^12,13^. In our study, colistin resistance in *A. baumannii* was attributed to the addition of pEtN to lipid A. The colistin-resistant mutant derived from the WT strain exhibited several amino acid alterations in the *pmrCAB* operon, but not in the *lpxACD* operon. On the contrary, both the colistin-dependent mutant and its colistin-resistant derivative had a disruption (IS*Aba1* insertion) in the *lpxC* gene, which encodes the UDP-3-O-acyl-N-acetylglucosamine deacetylase and plays an essential role in the biosynthesis of lipid A^18^. Further, the *pmrCAB*, *lpxA*, and *lpxC* genes were overexpressed in the colistin-resistant strain (H08-391R) derived from the WT strain, but not in the colistin-dependent strain and its derivative resistant strain. Additionally, MALDI-TOF MS analysis showed that both the colistin-dependent mutant and its colistin-resistant derivative lacked major peaks (*m*/*z* 1728 and *m*/*z* 1910) characteristic of lipid A, which were present in the spectra of lipid A from the colistin-susceptible WT strain and the colistin-resistant mutant derived *in vitro*. Although some peaks corresponding to lipid A were found in MS analysis of colistin-dependent strain, they may be remnants of lipid A during conversion into colistin dependence. Thus, we postulate that disruption in *lpxC* probably results in the loss of LPS or defects in its structure, which have an impact on the emergence of colistin dependence. Such LPS loss or structural defects would be preserved in the colistin-resistant mutant derived from the colistin-dependent mutant. However, the mechanisms that contribute to the survival of LPS-deficient *A. baumannii* and protection of the cell from colistin stress are unknown.

Although H08-391D and H08-391D-R had extremely high colistin MICs, they showed decreased MICs for most other antibiotics tested, except polymyxins. This is in concordance with previous studies with colistin-dependent *Acinetobacter* sp.^14,15^, but our findings that the colistin-resistant strain derived from the colistin-dependent mutant showed similar antibiotic susceptibilities as the latter is novel. It is well known that the bacterial outer membrane acts as a selective permeability barrier between the cytoplasm and the outside environment^19,20^. The outer membrane fragility in colistin-dependent *A. baumannii* may have contributed to its increased susceptibility to other antibiotics. In addition, the increased susceptibility to other antibiotics in the colistin-dependent mutant and its colistin-resistant derivative may provide new insights for the development of clinical therapeutics against *A. baumannii* infections.

In conclusion, our work reveals that colistin dependence in *A. baumannii* may be a transient phenotype, and might convert to an extremely resistant phenotype even when cultured in the antibiotic-free media. The colistin-dependent phenotype may arise from the loss of LPS or defects in its structure, resulting from the disruption of LpxC. However, we do not yet understand the comprehensive mechanisms of colistin dependence and its clinical implications. Thus, further investigations of the genetic basis of colistin dependence in *A. baumannii* are required.

## Materials and Methods

### Bacterial strains and growth conditions

Two colistin-susceptible *A. baumannii* strains, H08-391and H06-855, isolated from patients with bacteremia were used as the parental strains. Colistin-resistant subpopulations and colistin-dependent mutants were obtained from H08-391 and H06-855 through population analysis and colistin disc diffusion assay^16^. We then obtained the colistin-resistant mutants H08-391D-R and H06-855D-R from H08-391D and H06-855D, respectively, by serial passaging in drug-free medium. Briefly, we picked three or four colistin-dependent colonies surviving near the colistin disc, and cultured them overnight. Overnight cultures of the dependent mutants were diluted into fresh LB medium (1:100) without colistin, and incubated with vigorous shaking at 37 °C for 24 h. The MICs of colistin were estimated using the broth microdilution method during the course of each passage, for all serial transfer cultures and colistin disc diffusion assay were performed. Bacteria were routinely grown in the CA-MH media, with or without 10 mg/L colistin, at 37 °C.

### Determination of antimicrobial susceptibility

The MICs of 16 antimicrobial agents, including colistin, polymyxin B, imipenem, meropenem, tigecycline, tetracycline, ciprofloxacin, amikacin, cefepime, ceftriaxone, ceftazidime, piperacillin/tazobactam, ampicillin/sulbactam, rifampin, azithromycin, and vancomycin were evaluated using the standard broth microdilution method according to the CLSI guidelines M100-S25^17^. For the colistin-dependent mutants, H08-391D and H06-855D, colistin MICs were determined using concentrations ranging from 5 to 5120 mg/L. The MIC was defined as the lowest antibiotic concentration that yielded no visible growth. *Escherichia coli* ATCC 25922 and *Pseudomonas aeruginosa* ATCC 27853 were employed as control strains. The MIC values were confirmed by three independent experiments. To verify colistin resistance and dependence, the colistin disc diffusion assay was performed as previously described^21^.

### Sequencing of *pmrCAB*, *lpxC*, *lpxA*, and *lpxD* genes

The *pmrCAB*, *lpxC*, *lpxA*, and *lpxD* gene sequences (ACICU_03004, ACICU_03003, ACICU_03002, ACICU_03528, ACICU_02088, and ACICU_02090 in *A. baumannii* ACICU, respectively) were determined using the primers listed in Table 4. The sequences were analyzed using the DNASTAR software (DNASTAR Inc., Madison, WI, USA). The amino acid sequences of the WT strain, H08-391, and its derivatives were compared with those of the reference strain, *A. baumannii* ACICU (GenBank accession number: CP000863).

**Table 4:** Oligonucleotides used in this study.

**Primer**	**Sequence (5′ → 3′)**	**Amplicon size (bp)**	**Reference**
**Primers for sequencing**			
pmrC-F	CGGTAAGCGTGATACCTTTGA	1966	This study
pmrC-IF	CCGTGGTCGGTGTTTTACTT		
pmrC-R	GAGCCAAACCATCTAAACCGT		
pmrA-F	ACTGGACATGTTGCACTCTT	852	This study
pmrA-R	TGAAGTGCAACCTTATAAGCAC		
pmrB-F	ATTCGAACCATCCGAGGACT	1504	This study
pmrB-R	TGCGAGGAGCACATTTTCTA		
lpxA-F	TGAAGCATTAGCTCAAGTTT	1179	(12)
lpxA-R	GTCAGCAAATCAATACAAGA		
lpxD-F	CAAAGTATGAATACAACTTTTGAG	1483	(12)
lpxD-R	TTGAGCTAATGCTTCAACAA		
lpxC-F	TGAAGATGATGTTCCTGCAA	1504	(12)
lpxC-R	TGGTGAAAATCAGGCAATGAA		
**Primers for qRT-PCR**			
rpoB-QF	ATGCCGCCTGAAAAAGTAAC	154	(22)
rpoB-QR	TCCGCACGTAAAGTAGGAAC		
pmrC-QF	TGGAAATGGACTGCCAAAAT	137	This study
pmrC-QR	ACTTCCGAAACATCGGTCTG		
lpxA-QF	GTGGGGCTTCTTTGATCCTT	139	This study
lpxA-QR	CCAACCTTTTCTTCGCATACC		
lpxC-QF	GTGAGGCACGAACTTTTGGT	107	This study
lpxC-QR	TCGGCAAATCGTAATCCTTC		

### Quantitative RT-PCR (qRT-PCR)

Expression levels of the *pmrCAB* operon, *lpxA*, *lpxD*, and *lpxC* were determined by qRT-PCR. Total RNA was extracted from mid-log phase bacterial cultures (optical density at 600 nm [OD_600_]: ~0.5) using the Qiagen RNeasy Mini Kit (Qiagen, Hilden, Germany) according to the manufacturer’s instructions. Contaminating genomic DNA was removed from the RNA samples using the Ambion DNA-free kit (Invitrogen, Carlsbad, CA, USA), in accordance with the manufacturer’s protocol. Purified RNA was quantified spectrophotometrically. Reverse transcription reactions were performed using the Omniscript Reverse Transcriptase (Qiagen), per manufacturer’s protocol. To quantify the target genes, qRT-PCR was performed using the SYBR Green PCR Master Mix (Applied Biosystems, Foster City, CA, USA) on the QuantStudio 6 Flex Real-Time PCR System (Applied Biosystems, Foster City, CA, USA). The primers for this study were designed using Primer 3 (Table 4). The fold-changes were calculated according to the threshold cycle (CT) method, using the *rpoB* gene, which encodes the β subunit of bacterial RNA polymerase, as the reference^22^. The experiments were repeated with three independent cultures, each tested in duplicate. Differences in the expression levels of the target genes were analyzed by Student’s unpaired *t*-test using GraphPad Prism 5. Differences were considered to be significant at P < 0.05.

### Transcriptomic analysis (mRNA sequencing)

Overnight culture of *A. baumannii* H08-391, H08-391D, H06-855 and H06-855D was diluted into fresh LB broth (1:100) and then the culture was incubated with vigorous shaking (250 rpm) at 37 °C until the OD600 reaches 0.5-0.6. Total RNA extracted using the Qiagen RNeasy Mini Kit (Qiagen, Hilden, Germany). After isolation of RNA, cDNA was synthesized and sequencing libraries were generated in strand-specific manner according to the Illumine standard protocol for high-throughput sequencing. Library sequencing was performed with Illumina HiSeq. 2000 sequencing system (Illumina, USA) at Macrogen Inc. (Seoul, Korea). Raw reads were mapped to the reference genome sequence of *A. baumannii* ACICU (GenBank accession No. CP000863.1). Expression levels of mRNA were expressed as reads per kilobase per million sequence reads (RPKM), which considers the effect of sequencing depth and gene length for the reads.

### Lipid A isolation and structural analysis

For structural analysis, lipid A was extracted by an ammonium hydroxide-isobutyric acid method as previously described^23^, and subjected to MALDI-TOF MS analysis. Briefly, freshly washed cells (10 mg) were suspended in 400 μL of isobutyric acid-1M ammonium hydroxide mixture (5:3, vol/vol), and incubated for 2 h at 100 °C in a screw-cap test tube, with occasional vortexing. The mixture was then cooled on ice and centrifuged at 8000 g for 15 min. The supernatant was transferred to a new tube, mixed with an equal volume of water, and lyophilized overnight at −70 °C. The lyophilized sample was then washed twice with 400 μL of methanol, and centrifuged at 5000 rpm for 15 min. Finally, the insoluble lipid A was solubilized in 100 μL chloroform-methanol-water mixture (3:1.5:0.25, v/v/v). The lipid A structure was analyzed using MALDI-TOF mass spectrometry in the negative-ion mode^8,24^. All MALDI-TOF analyses were performed on a Bruker Ultraflex III TOF/TOF mass spectrometer (Bruker Daltonics, Coventry, UK) using the FlexControl 3.0 acquisition software. The matrix used for lipid A analysis was 2,5-dihydroxybenzoic acid (DHB; Sigma Chemical Co., St. Louis, MO, USA). The DHB solution (10 mg/mL) was prepared using a mixture of water and acetonitrile (1:4, vol/vol). All lipid A samples were premixed with the DHB solution (1:1, vol/vol) before MALDI measurements, and 1 μL of the resulting mixture was spotted on the MALDI metallic target. Lipid A from *E. coli* F583 was used as an external standard and mass calibrant.

### Extraction and visualization of LPS

For the LPS extraction, we used a hot aqueous-phenol method as previously described with some modifications^25^. In brief, 1.5 ml bacterial suspensions (OD600 of 0.5) were centrifuged and the pellets were resuspended in 200 μL SDS-lysis buffer (2% β-mercaptoethanol, 2% SDS and 10% glycerol in 50 mM Tris-HCL, pH 6.8) and boiled for 15 minutes. In order to eliminate contaminating protein and nucleic acids, proteinase K, DNase and RNase was subsequently treated. Then, an equal volume of ice-cold Tris-saturated phenol was added and the mixtures were incubated at 65 °C for 15 minutes with vortexing occasionally. At the next step, 1 mL of diethyl ether were added to the mixtures and centrifuged at 20000 × *g* for 10 minutes. The bottom layer was extracted and the final purified LPS product was separated by SDS-PAGE. Silver staining of the gels was performed according to the standard protocol.

### Membrane potential assay

Membrane potential was measured using the BacLight Bacterial Membrane Potential Kit (Molecular Probes, Invitrogen, Carlsbad, CA, USA) and the xMark Microplate Absorbance Spectrophotometer (Bio-Rad, Irvine, CA, USA) according to the manufacturer’s protocol. In brief, overnight cultures were diluted 1:100 in fresh LB medium and grown to the exponential phase (OD_600_: 0.5). The bacterial cultures were diluted to approximately 1 × 10^6^ cells/mL in filtered phosphate-buffered saline (PBS). Three aliquots of 1 mL each of the bacterial suspension were prepared for the test sample, depolarized control, and unstained control. To the depolarized control sample, 10 μL of 500 μM CCCP was added. Then, 10 μL of 3 mM DiOC_2_(3) was added to all tubes except that of the unstained control, and incubated for 10 min at 24 °C. The ratio of the red (Ex. 530 nm/Em. 590 nm) and green (Ex. 485 nm/Em. 528 nm) fluorescence intensities was used as an indicator of the membrane potential. Data were analyzed using the Microplate Manager Software 6.0. The experiments were repeated with three independent cultures, each tested in triplicate.

## Electronic supplementary material

Figure S1
