# Supplementary material for: Transition of colistin dependence into colistin resistance in Acinetobacter baumannii
Source: Sci Rep. 2017 Oct 27;7:14216. doi: 10.1038/s41598-017-14609-0 (PMC5660220; doi:10.1038/s41598-017-14609-0)

**Transition of colistin dependence into colistin resistance in *Acinetobacter baumannii***

**Ji-Young Lee, Eun Seon Chung, and Kwan Soo Ko***

Department of Molecular Cell Biology, Sungkyunkwan University School of Medicine, Suwon 16419, South Korea

**Supplementary Figure 1.** Conversion of colistin dependence into complete colistin resistance in the strains: H06-855 and H09-146.


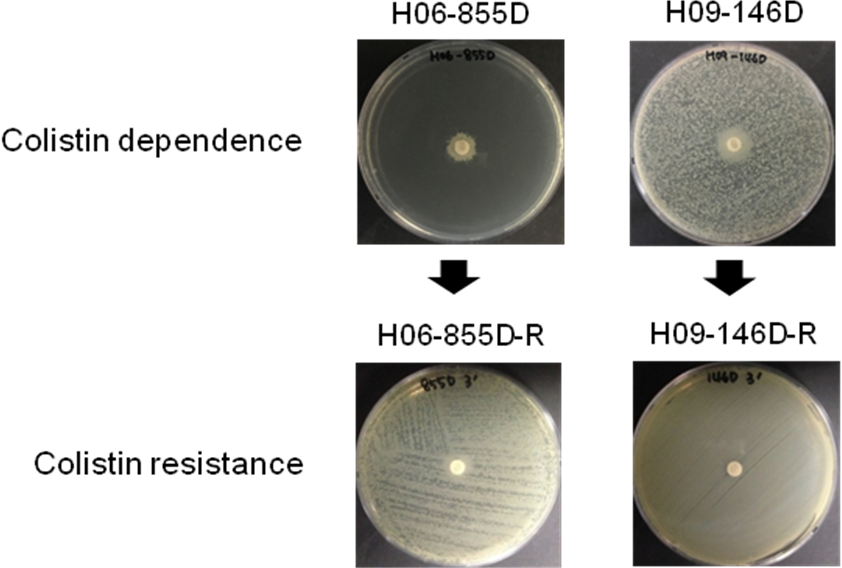

Supplement: Supplementary file 1 — Figure S1 [file 41598_2017_14609_MOESM1_ESM.doc]
